# Anthropometry and diagnostic aware deep learning for exercise assessment

**DOI:** 10.3389/fmedt.2025.1725661

**Published:** 2026-02-06

**Authors:** Karla Miriam Reyes Leiva, Pavla Nikelova, Martin Cerny

**Affiliations:** Faculty of Electrical Engineering and Computer Science, VSB Technical University of Ostrava, Ostrava, Czech Republic

**Keywords:** anthropometry, biomechanics, deeplearning, exercise assessment, personalized AI, rehabilitation, wearable sensors

## Abstract

**Background:**

Correct technique during strength exercises such as squats and Romanian deadlifts (RDLs) is fundamental for performance and injury prevention.

**Objective:**

We introduce ADA (Anthropometry and Diagnostic Aware), a multimodal deep-learning framework that integrates IMU kinematics with anthropometric and diagnostic features to classify movement quality and predict movement related risk.

**Methods:**

Seventeen-sensor IMU data were collected from 15 healthy subjects performing correct and incorrect squat and RDL trials. A CNN-LSTM branch processed kinematic sequences and a fully connected branch processed static anthropometric/diagnostic inputs; feature fusion used attention weighting.

**Results:**

Incorporating anthropometry and diagnostic context increased sequence-level accuracy from 86.5% (kinematics only) to 94.8% (ADA) and enabled binary risk prediction at 97.8%. Personalized (transfer learning) fine tuning further improved accuracies (mean gains 3%–5% depending on window length).

**Conclusion:**

ADA demonstrates that subject-specific static features improve movement quality classification and risk stratification, supporting wearable-based personalized feedback in training and rehabilitation.

## 1 Introduction

The squat is a fundamental movement pattern and a cornerstone of strength and conditioning programs, widely used to improve the strength of the lower extremities, trunk stability, and overall functional capacity (1). As a compound, multi-joint exercise involving the hips, knees, and ankles, its execution directly influences biomechanical loading, muscle activation, and injury risk (2). Squats are incorporated across various disciplines, including powerlifting, CrossFit, and rehabilitation protocols, where correct technique is essential for performance optimization and injury prevention (3). Despite its apparent simplicity, the squat requires coordinated activation of multiple muscle groups, with variations in depth, stance, and load placement leading to distinct kinematic and kinetic profiles (4).

To analyze these complex movement patterns, Motion Capture (MoCap) technologies have long been considered the gold standard for quantifying joint kinematics with high spatial and temporal precision (5, 6). Both marker-based optical systems and IMU-based wearable sensors have been employed to measure joint angles, segment trajectories, and compensatory strategies during squatting (7). Squat execution is further influenced by anthropometry, training experience, and external constraints (8, 9), underlining the importance of subject-specific biomechanical context in performance evaluation.

In recent years, Human Activity Recognition (HAR) frameworks have increasingly integrated machine learning and **deep learning** techniques with MoCap and IMU data to automatically assess movement quality and detect deviations from ideal technique (10, 11, 24). Hybrid architectures such as Convolutional Neural Networks combined with Long Short-Term Memory (CNN–LSTM) networks have demonstrated superior performance in recognizing dynamic exercise patterns (12). Further optimizations in windowing (12), computational complexity, and reduced-sensor configurations (11) have demonstrated the feasibility of scalable, real-time feedback systems for both training and **rehabilitation**. Nevertheless, most studies address either biomechanical analysis or automated classification in isolation, rarely combining detailed kinematic evaluation with deep learning-based movement assessment. Moreover, the influence of individual **anthropometric** characteristics on exercise classification remains largely unexplored, despite evidence that body proportions significantly affect squat mechanics (13).

The state of the art in squat biomechanics research is thus characterized by two main directions: high-resolution kinematic/kinetic characterization and AI-driven movement classification. Recent studies have clarified how technique parameters such as stance width influence joint loading and muscular force generation. For example, McMahon et al. found that narrower stances enhance quadriceps force and peak power, whereas wider stances increase posterior-chain activation and medial ground reaction forces (14). Similarly, Larsen, de Zee, and van den Tillaar reported that wider stances in heavy back squats reduce knee flexion angles and extension moments while increasing hip abduction, highlighting important biomechanical trade-offs (15).

Complementing these biomechanical insights, AI approaches leveraging IMU data have achieved significant progress. Zhang et al. (16) proposed a hybrid CNN–GRU model trained on wearable sensor data from squats, lunges, and jumps, achieving robust classification accuracy across sampling rates. Likewise, (17) introduced a deep learning framework for estimating lower-limb joint kinematics from IMUs, incorporating transfer learning to adapt models to new subjects with minimal data, and identifying femur and calcaneus sensors as optimal placements for accurate predictions (18, 19). These advances illustrate the growing potential of AI-based motion analysis, yet few frameworks integrate user-specific biomechanical or diagnostic factors.

Despite this progress, most existing AI systems treat subjects as biomechanically homogeneous and overlook inter-individual variations in body structure, flexibility, or clinical conditions. This omission limits their applicability in real-world, field-deployable contexts where personalization is critical for valid exercise assessment and injury risk evaluation. As wearable-based movement monitoring expands toward personalized training and rehabilitation, incorporating subject-specific context has become increasingly essential.

The proposed ADA (Anthropometry and Diagnostic Aware) framework directly addresses these gaps by fusing individualized anthropometric dimensions and diagnostic features with IMU-derived kinematic sequences. ADA employs a multimodal deep learning network that combines temporal motion features with static subject characteristics through attention based feature fusion, enhancing both the accuracy and interpretability of personalized AI driven movement classification.

Specifically, this study aims to:

Quantify kinematic differences between correctly and incorrectly executed squats and Romanian deadlifts (RDLs) using wearable MoCap systems.
Examine the influence of anthropometric characteristics on squat execution parameters and their detectability by machine learning models.
Develop an anthropometry and diagnostic aware AI model to classify squat and RDL movement quality (correct vs. incorrect) and highlight biomechanical and subject specific factors influencing performance.

By integrating wearable sensors, biomechanics, and deep learning within a subject specific modeling paradigm, ADA provides a scalable approach for personalized exercise assessment and rehabilitation, bridging the gap between laboratory grade biomechanical precision and real world applicability.

## 2 Materials and methods

The study quantified kinematic differences between correctly and incorrectly executed squats and RDLs using a wearable inertial motion-capture system (Xsens MVN 2019.2). The primary objectives were to (i) identify kinematic deviations between correct and incorrect technique and (ii) evaluate how subject specific anthropometric and diagnostic features improve automatic movement-quality classification and risk prediction. Ethical approval was obtained from the Ethics Committee of the Faculty of Electrical Engineering and Computer Science, VSB TUO.

### 2.1 Subjects

Fifteen healthy adult subjects (8 males, 7 females; age 19–35 years) were recruited. Inclusion criteria were absence of acute musculoskeletal injury and ability to perform deep squats. Each subject performed 40 repetitions in total: 10 correct squats, 10 incorrect squats, 10 correct RDLs and 10 incorrect RDLs. Anthropometric summary statistics are given in Table 1 (n=15). All subjects provided written informed consent prior to participation.

**Table 1:** Subject anthropometric characteristics.

Parameter	Mean ± SD	Range
Height (cm)	168.9 ± 7.6	153–180
Foot length (cm)	23.3 ± 1.6	21–26
Arm height (cm)${}^{a}$	137.1 ± 6.6	124–147
Shoulder width (cm)	37.5 ± 3.3	30–42
Arm span (cm)	170.1 ± 8.9	150–181
Hip height (cm)	95.3 ± 4.9	88–103
Hip width (cm)	33.5 ± 3.6	27–40
Knee height (cm)	46.2 ± 2.1	43–50
Ankle height (cm)	6.1 ± 1.1	5–9

${}^{a}$Arm height corresponds to acromion height measured in standing posture.

### 2.2 Instrumentation and setup

Full body kinematics were recorded with the Xsens MVN Link system using 17 IMUs placed according to the manufacturer’s protocol. Data were sampled at 60 Hz and streamed to the acquisition PC via the access point and MVN software. System calibration followed the vendor’s procedure prior to each session.

### 2.3 Experimental protocol

**Figure 1:**
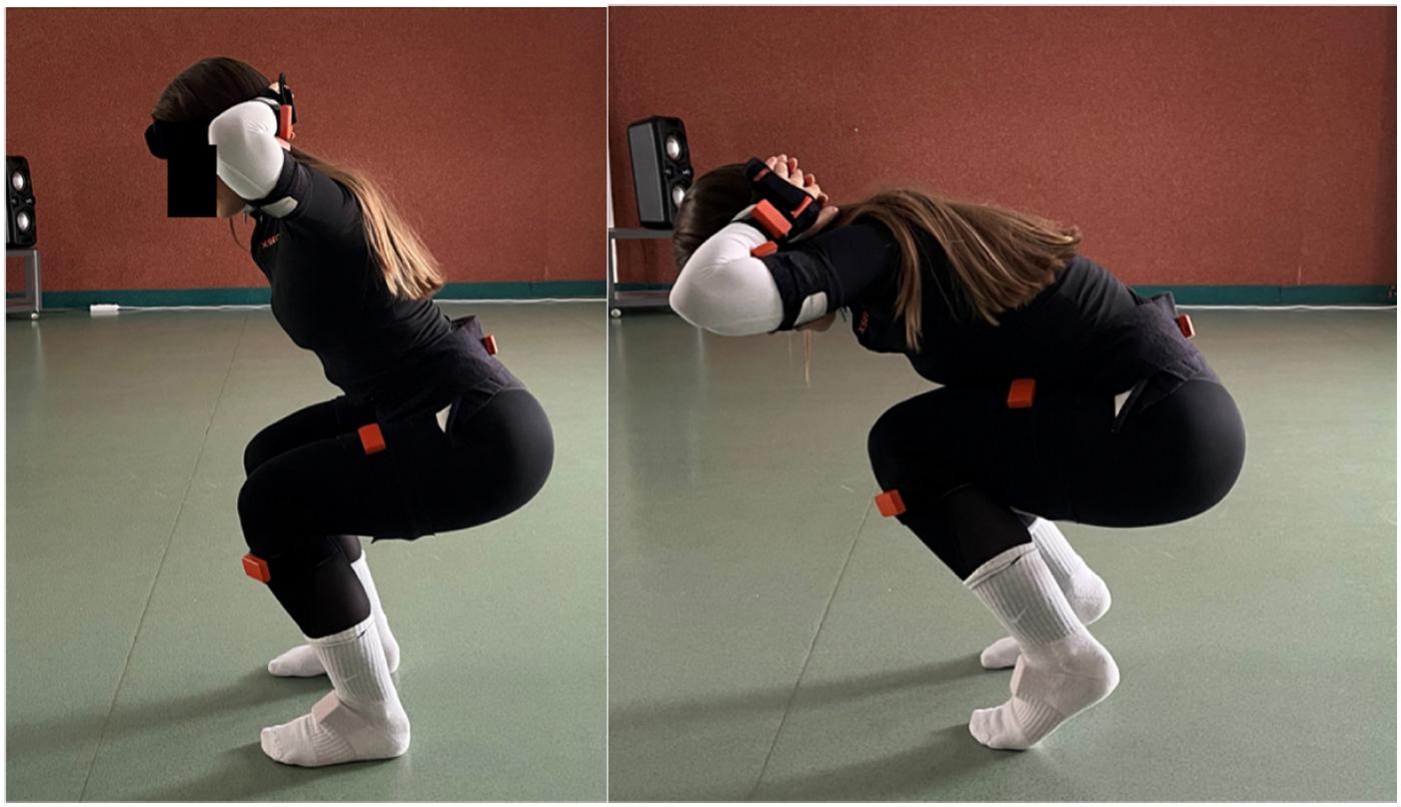
Comparison of correct vs. incorrect squat technique (side view).

Sessions began with a 5 min dynamic warm up. Under the supervision of a certified fitness trainer, each subject performed: 10 correct deep squats (knee flexion > 90${}^{\circ }$), 10 intentionally incorrect squats (e.g., heels lifted, excessive knee adduction, trunk flexion), 10 correct RDLs and 10 incorrect RDLs (e.g., knee hyperextension, excessive trunk flexion). Squats were executed with hands behind the head and RDLs with hands alongside the thighs. Total session duration per subject did not exceed one hour. Example correct/incorrect squat kinematics are illustrated in Figure 1.

### 2.4 Data processing and segmentation

**Figure 2:**
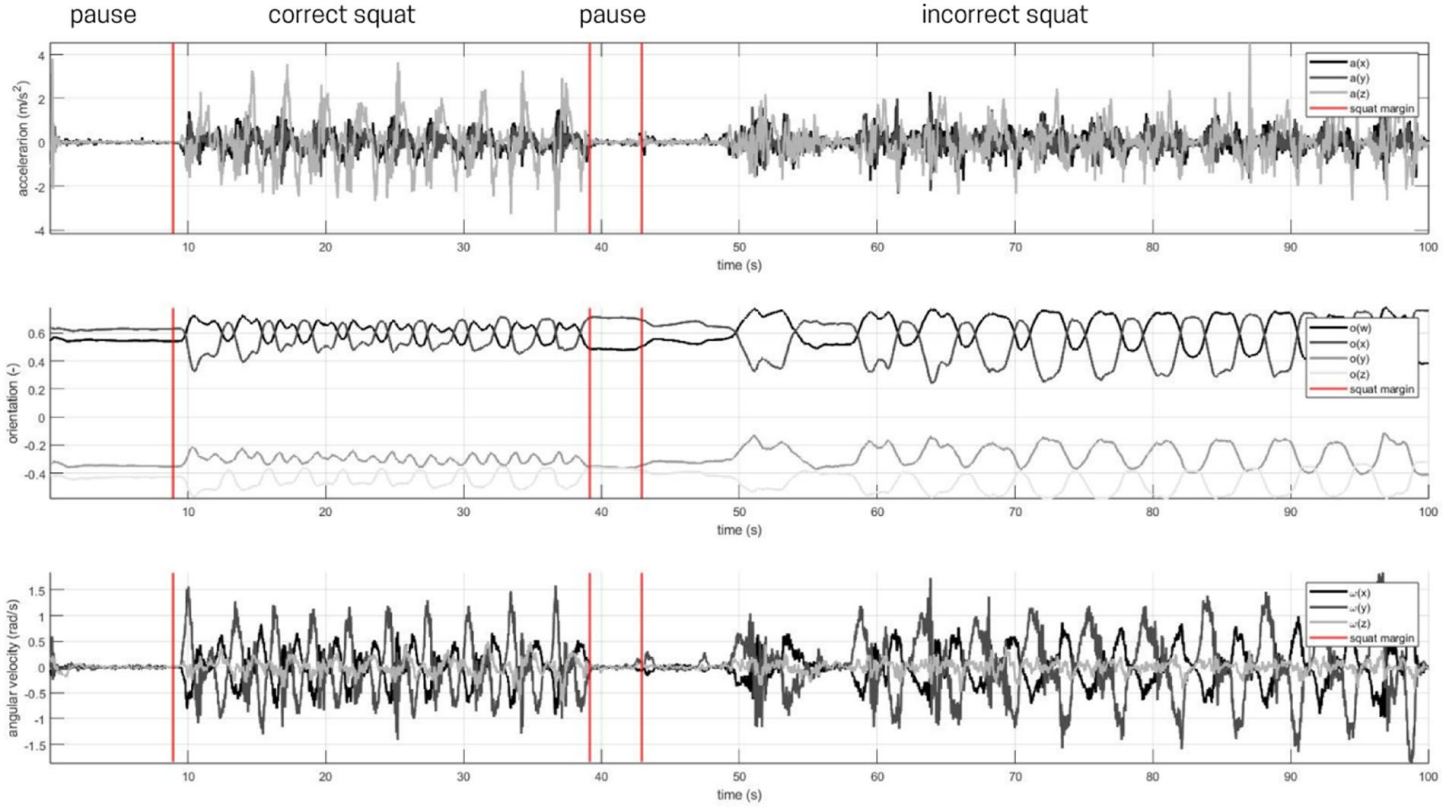
Example of annotated knee flexion signal showing correct (1), pause (0), and incorrect (2) phases.

Raw MVN outputs (quaternions, linear accelerations, angular velocities) were exported and processed in MATLAB. Joint angles (hip, knee, ankle flexion; knee adduction) were computed via quaternion to Euler conversions. Repetition segmentation combined the IMU derived kinematic signals with synchronized video to obtain reliable start/end markers; manual annotation was used to assign frame level labels (correct, incorrect, pause) see Figure 2 for annotated joint angle data. From each repetition we extracted overlapping sequences of 50–200 frames (windowing parameters described in Supplementary Material), resulting in approximately 1,500 labelled sequences in the dataset. Sequence-level labels were used for model training and evaluation; however, inferential statistical tests (*t*-tests, correlations) were performed on subject level summary measures (e.g., mean peak angle per subject) to avoid pseudoreplication.

### 2.5 Statistical analysis

Descriptive statistics (mean, SD) were computed for kinematic features and repetition durations. Normality was assessed with the Shapiro-Wilk test. Paired samples *t*-tests (or Wilcoxon signed rank tests when normality assumptions were violated) compared correct vs. incorrect executions within subjects; these tests used subject level summary statistics. Spearman rank correlations assessed relationships between anthropometric measures and joint ranges; primary correlations are reported with *p*-values and 95% bootstrap confidence intervals (1,000 resamples). Effect sizes for group comparisons are reported as Cohen’s d (paired) with 95% CIs where applicable. All inferential tests are presented as exploratory (no correction for multiple comparisons); this is noted in the Limitations.

### 2.6 Anthropometry aware model architecture and training

To account for inter subject variability, we combined sequence level kinematic inputs with standardized anthropometric features (z-scored). The multimodal network comprised: (i) a 1D convolutional front end followed by stacked LSTM layers to learn temporal kinematic representations, and (ii) a fully connected branch processing static anthropometric and diagnostic inputs. Feature-level attention fused the two branches before final classification. The network produced two outputs: movement-quality classification (binary: correct vs. incorrect) and a binary risk prediction.

Training used a two stage strategy: population level pretraining (generic model) followed by subject specific fine tuning (personalized model, transfer learning). Typical training hyperparameters were: Adam optimizer, initial learning rate $1\times 10^{-4}$, batch size 32, early stopping with patience = 10 epochs, and dropout regularization. Generic models were evaluated with 5-fold subject wise cross validation; personalized models were evaluated with a 3-fold subject wise cross validation (folds defined at subject level so no sequences from the same subject appear in both train and test sets).

### 2.7 Diagnostic label construction and clustering

Diagnostic variables (e.g., ankle dorsiflexion, hip mobility tests, prior musculoskeletal findings) were encoded and merged with anthropometrics. The binary risk label was defined as “high” if a subject presented ≥1 of: (i) documented prior lower limb surgery (e.g., ACL reconstruction), (ii) clinically reduced hip mobility (hip flexion < $100^{\circ }$ or hip internal rotation < $25^{\circ }$), or (iii) ankle dorsiflexion < $15^{\circ }$ (weight-bearing lunge test); otherwise the label was “low.” In this dataset 4 subjects met at least one risk criterion (high risk) and 11 subjects were low risk; after segmentation this corresponded to high = 432 and low = 1068 sequences.

Latent features from the penultimate layer were standardized (*z*-score) prior to unsupervised analysis. t-SNE was used for 2D visualization and *k*-means (k=3) for exploratory grouping. Cluster validity was quantified with the silhouette coefficient (mean silhouette ≈ 0.46), indicating moderate separation between clusters.

### 2.8 Performance evaluation

Model performance was assessed at sequence level. For each fold we computed accuracy, precision, recall, F1-score and area under the ROC curve (AUC). Reported metric values are the mean ± SD across cross-validation folds. AUCs were computed from predicted probabilities for the positive class and averaged across folds. Confusion matrices reported in Results are aggregated across folds (sum of fold level matrices). To compare generic and personalized model accuracies we used paired samples t-tests on fold wise accuracies (or Wilcoxon signed rank tests when normality was violated). Per fold accuracy vectors and analysis scripts are provided in the Supplementary Material to allow reproducibility.

All analyses were performed in MATLAB and R (RStudio); machine learning models were implemented in Python (TensorFlow/Keras). Significance was assessed at α=0.05 unless otherwise stated, and all statistical analyses are reported as exploratory.

## 3 Results

### 3.1 Anthropometric and kinematic findings

**Figure 3:**
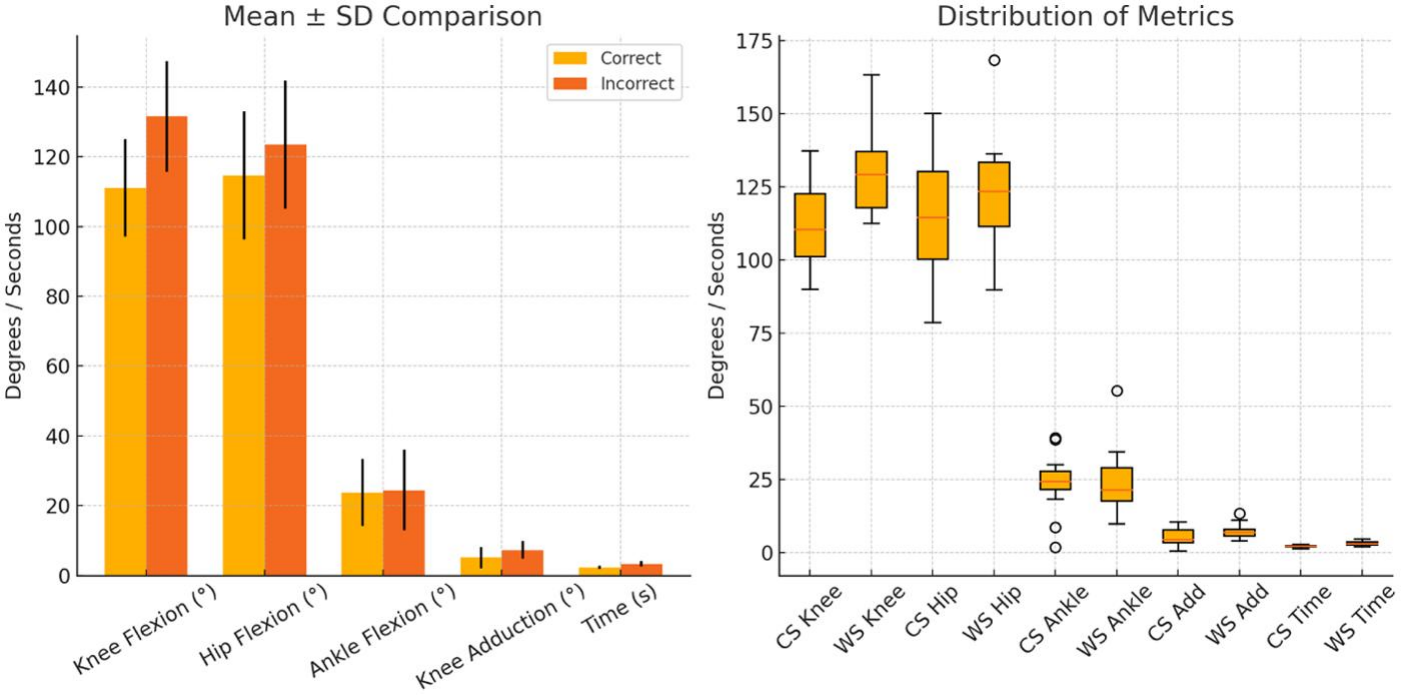
Group-level summary of correct vs. incorrect squat kinematics. Error bars represent ±1 SD.

Subjects had a mean height of 168.9 ± 7.6 cm, with segment lengths consistent with expected adult proportions. Clear kinematic differences were observed between correct and incorrect squat executions (Figure 3). Incorrect squats showed greater knee flexion (131.5 ± $15.8^{\circ }$ vs. 111.1 ± $14.0^{\circ }$) and hip flexion (123.5 ± $18.4^{\circ }$ vs. 114.6 ± $18.4^{\circ }$), as well as increased knee adduction (7.3 ± $2.6^{\circ }$ vs. 5.1 ± $3.0^{\circ }$). All joint angle values reported correspond to peak flexion extracted from each repetition and averaged at the subject level. Execution time was also longer in incorrect squats (mean difference = 1.0 s; 95% CI: [0.6, 1.4], p<0.001) as shown in Figure 3. All comparisons were conducted on subject-level summaries using paired analyses.

**Figure 4:**
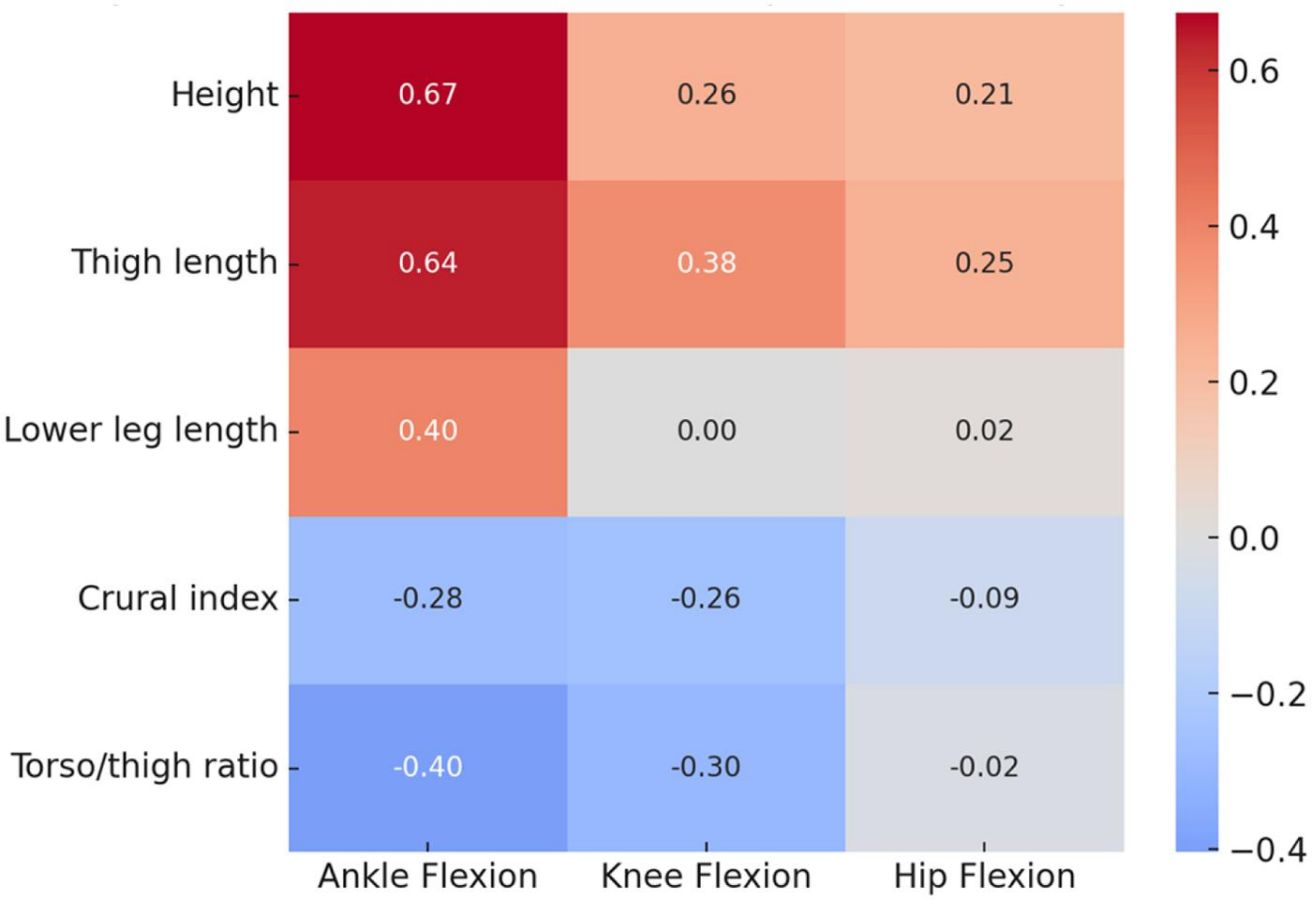
Heatmap of Spearman correlations between anthropometric parameters and joint flexion angles.

Shapiro–Wilk tests indicated no evidence of non-normality for these group-level summary measures (p>0.05), including ankle flexion in incorrect squats (p=0.087). Spearman correlations revealed that height (ρ=0.674, p=0.006) and thigh length (ρ=0.637, p=0.010) were positively associated with maximal ankle dorsiflexion, whereas correlations with maximal knee and hip flexion were not statistically significant (Table 2; Figure 4). These results suggest that distal joint mobility may be more influenced by anthropometric variation than proximal joint angles during squatting.

**Table 2:** Spearman correlations between body proportions and maximum joint flexion.

Body parameter	Ankle flexion ρ	Knee flexion ρ	Hip flexion ρ
Height	0.674 (0.006)	0.255 (0.360)	0.212 (0.449)
Thigh length	0.637 (0.010)	0.376 (0.168)	0.250 (0.369)
Lower leg length	0.404 (0.134)	0.004 (0.990)	0.020 (0.943)
Crural index	−0.277 (0.317)	−0.256 (0.358)	−0.086 (0.761)
Torso/thigh ratio	−0.404 (0.135)	−0.298 (0.280)	−0.021 (0.940)

### 3.2 Movement quality classification

The baseline kinematics only model achieved a mean accuracy of 86.5% and an AUC of 0.92. Incorporating anthropometric and diagnostic variables substantially improved performance: the multimodal ADA model reached 94.8% accuracy, with increases across all evaluated metrics (precision = 0.945, recall = 0.953, F1 = 0.949; Table 3). Confusion matrices aggregated across folds indicated more balanced and robust classification in ADA compared with the baseline.

**Table 3:** Classification performance for movement-quality prediction (mean ± SD across folds).

Model	Accuracy (%)	Precision	Recall	F1-score	AUC
Baseline (kinematics-only)	86.5 ± 3.0	0.864	0.867	0.865	0.92
ADA (multimodal)	94.8 ± 1.6	0.945	0.953	0.949	0.98

Accuracy across temporal input lengths (MAX_T = 50, 100, 150, 200 frames) is shown in Table 4. Personalized (subject-specific) models consistently outperformed generic models for all sequence lengths. Accuracy gains ranged from +3.3% to +4.5%, with 95% bootstrap confidence intervals indicating stable improvements across folds.

**Table 4:** Cross validation accuracy (mean ± SD) for generic and personalized models across sequence lengths.

MAX_T	Generic mean	Generic SD	Personalized mean	Personalized SD
50	0.8426	0.0138	0.9212	0.0443
100	0.8304	0.0477	0.8948	0.0655
150	0.7688	0.0847	0.9157	0.0398
200	0.7060	0.0596	0.7214	0.1395

**Figure 5:**
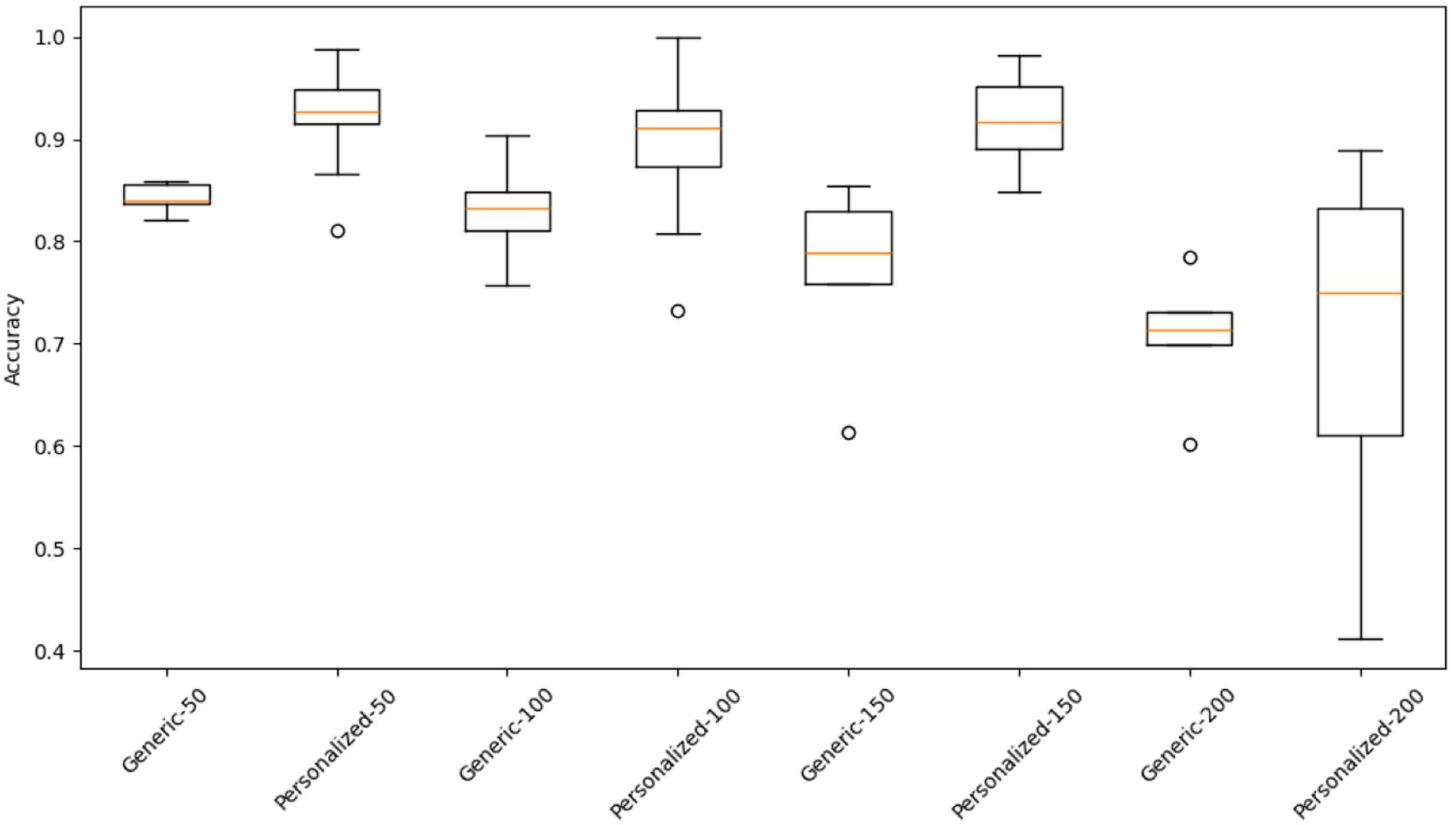
Fold-wise accuracy distributions for generic and personalized models across temporal input lengths.

Paired comparisons of fold wise accuracies confirmed statistically significant advantages for personalized over generic models (Table 5). Effect sizes were large (paired Cohen’s d = 0.86–1.73), indicating that personalization contributed meaningfully to inter-subject variability reduction regardless of input-window length. Figure 5 illustrates these improvements and their reduced variance.

**Table 5:** Paired samples *t*-tests (df = 4) comparing generic vs. personalized model accuracy across sequence lengths.

MAX_T	Mean diff (%)	t(4)	*p*-value	Cohen’s d
50	+3.9	4.90	0.007	1.10
100	+3.8	5.33	0.005	1.20
150	+4.5	7.74	0.001	1.73
200	+3.3	3.87	0.017	0.86

### 3.3 Diagnostic aware risk prediction

The ADA risk prediction branch achieved an accuracy of 97.8% across 1,500 sequences low = 1068, high = 432. High risk classification showed excellent sensitivity (recall = 0.984), precision (0.942) and F1-score (0.962), with an AUC of 0.99 (Table 6). These results demonstrate that static diagnostic and anthropometric information meaningfully enhances detection of movement-related risk beyond kinematic data alone.

**Table 6:** Diagnostic aware risk prediction performance and class distribution (total = 1,500 sequences).

Metric	High risk	Low risk
Support (*n*)	432	1,068
Precision	0.942	0.976
Recall (Sensitivity)	0.984	0.976
F1-score	0.962	0.976
Overall Accuracy = 97.8%, AUC = 0.99		

### 3.4 Latent space structure and subject stratification

To characterize subject specific biomechanical patterns, latent features extracted from the penultimate layer were projected using t-SNE and clustered with *k*-means (k=3). Anthropometric inputs were standardized prior to clustering. Silhouette analysis yielded a mean value of 0.46, indicating moderate cluster separability. The three clusters captured distinct biomechanical diagnostic profiles:

**Cluster 0 (*n* = 520)**: subjects with typical anthropometry and no diagnostic flags; dominated by low-risk, correct executions.
**Cluster 1 (*n* = 610)**: subjects exhibiting altered joint mobility (e.g., reduced hip rotation or ankle dorsiflexion) or minor musculoskeletal history; mixed-risk sequences.
**Cluster 2 (*n* = 370)**: subjects with multiple diagnostic findings and non-standard segmental proportions; enriched for high-risk and mechanically suboptimal executions.

**Figure 6:**
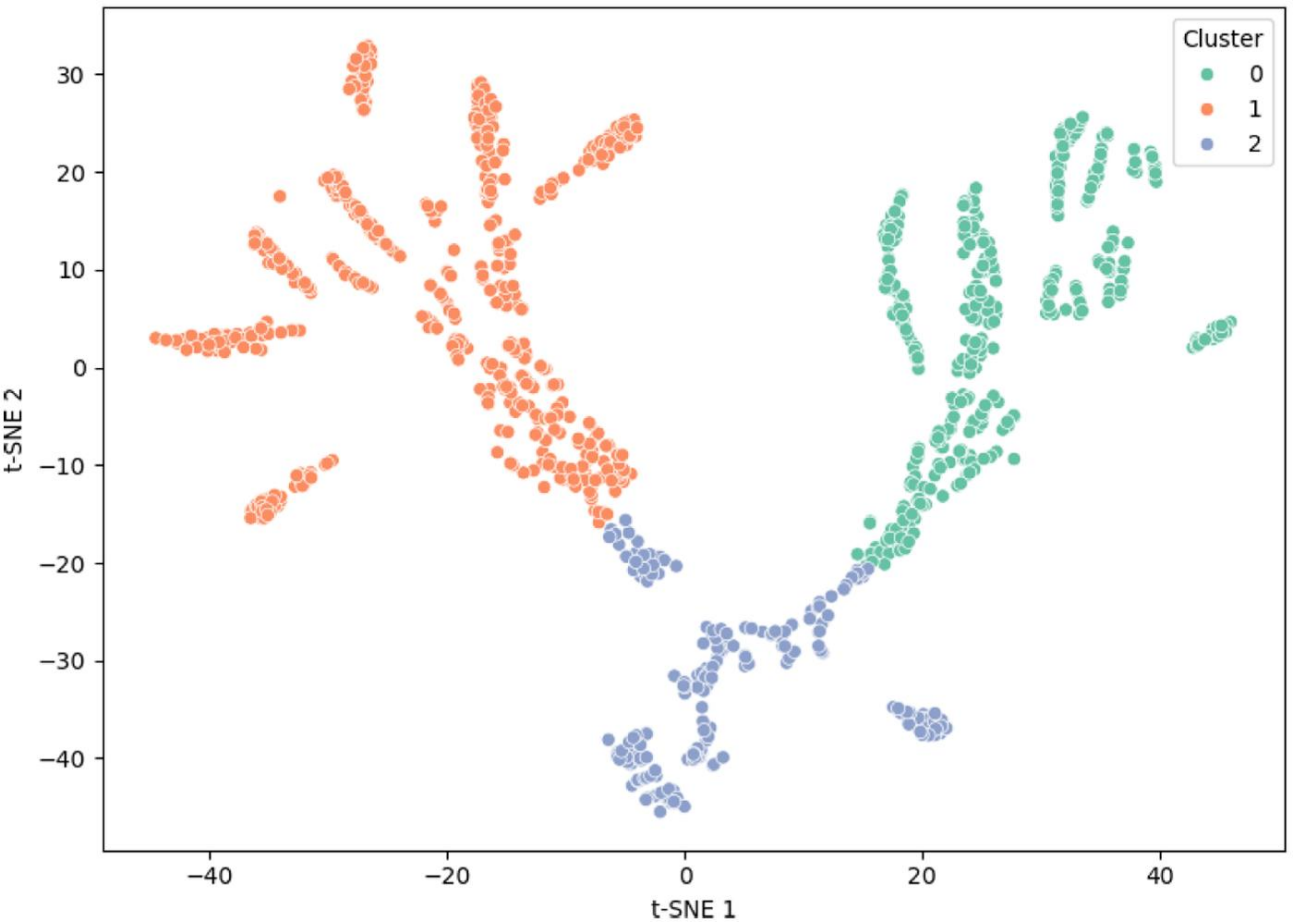
t-SNE projection of latent features colored by *k*-means cluster membership (k=3).

**Figure 7:**
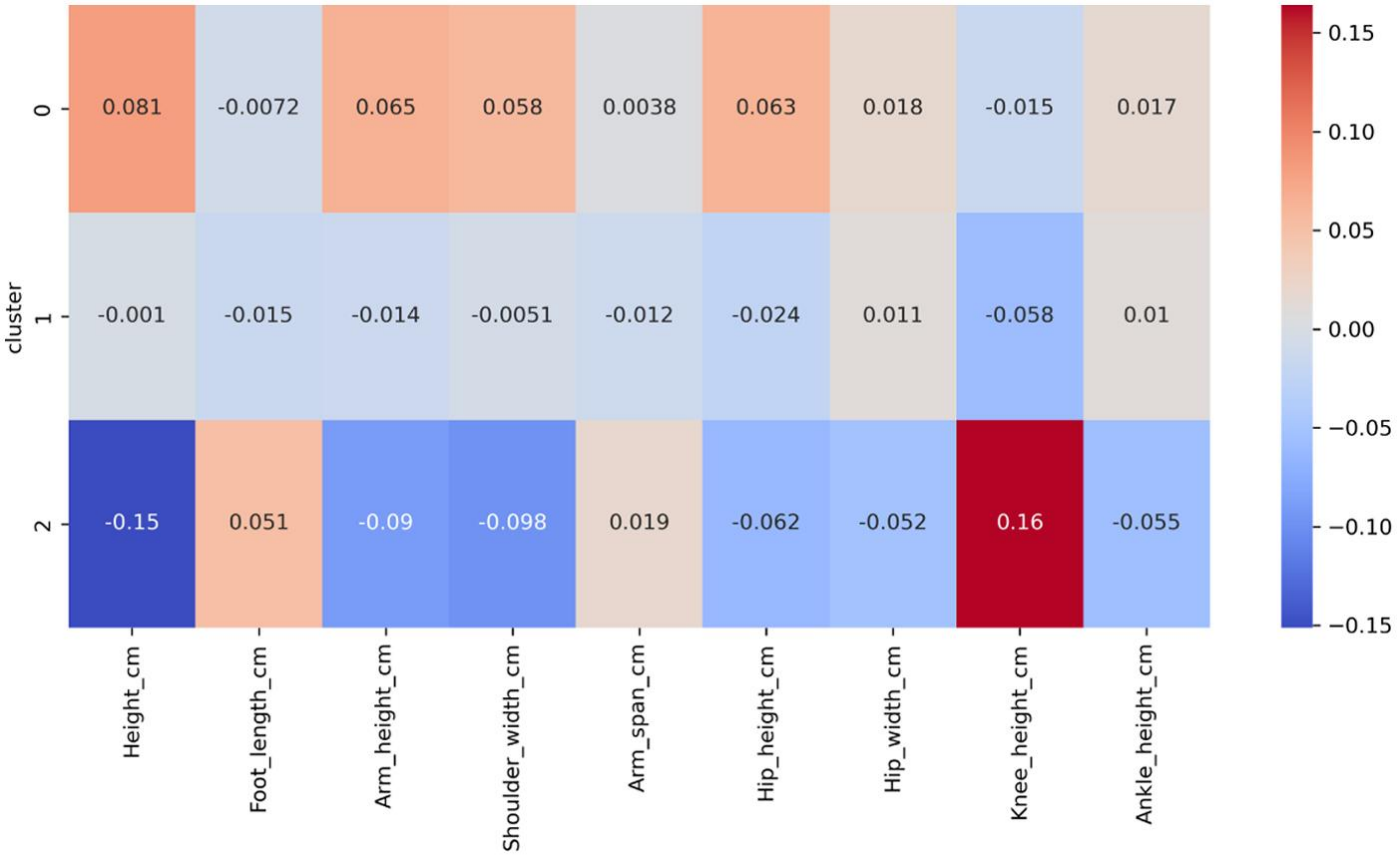
Standardized anthropometric feature profiles across the three clusters.

Figures 6, 7 illustrate latent-space organization and cluster-level anthropometric profiles, respectively. These clusters should be interpreted as exploratory groupings rather than diagnostic categories; however, the observed structure supports the rationale for integrating static subject-specific data into movement assessment.

Overall, incorrect squat technique produced substantial and consistent kinematic deviations. Anthropometric effects were most pronounced at the ankle, reflecting segment length influences on mobility. The ADA multimodal framework markedly improved movement-quality classification and enabled highly accurate risk prediction. Personalized fine-tuning provided additional gains, and exploratory clustering of latent features revealed interpretable biomechanical structure aligned with subject level characteristics. All statistical findings are reported as exploratory with effect sizes and confidence intervals to support transparent interpretation.

## 4 Discussion

This study evaluated whether integrating subject specific anthropometric and diagnostic information enhances IMU-based assessment of squat and RDL execution. All statistical analyses were exploratory and descriptive, with effect sizes reported alongside *p*-values; no corrections for multiple comparisons were applied. Findings should therefore be interpreted with caution regarding potential Type I error inflation.

### 4.1 Interpretation of principal findings

The results demonstrate that multimodal modeling substantially improves movement-quality classification compared with kinematics only approaches. The ADA framework outperformed the baseline model across all evaluation metrics, and personalized fine tuning yielded further gains. These findings align with recent work showing that subject aware architectures and adaptive attention mechanisms improve accuracy in human motion analysis. For example, Weng et al. (20) achieved 94.7% squat assessment accuracy using attention enhanced temporal CNNs, and Chen & Fan (21) reported notable improvements in posture recognition and physiological estimation when incorporating individualized model components. Together with the present results, this growing evidence indicates that anthropometric and diagnostic features offer meaningful subject level information not captured by kinematics alone.

The improvements observed in personalized models are consistent with broader trends in rehabilitation and sports AI research emphasizing the value of fine tuning for inter-subject variability. As highlighted by Zhang et al. (16), transfer learning facilitates generalization while maintaining interpretability, which is essential when applying AI to heterogeneous clinical or athletic populations. Our observed accuracy gains at shorter sequence lengths (e.g., 92.1% vs. 84.3% at 50-frame inputs) illustrate the practical relevance of personalization, especially when only limited subject data are available.

The clustering analysis further supports the utility of multimodal feature integration. Latent representations revealed three biomechanically interpretable profiles structured by anthropometric proportions and diagnostic findings. These exploratory clusters echo previous evidence that physiologically grounded features or synthetic augmentation improve subject stratification (22). Although not intended as diagnostic categories, the clusters contribute to the interpretability of the ADA framework by demonstrating that the network organizes subjects according to meaningful biomechanical and clinical characteristics.

Finally, our findings resonate with broader trends in wearable AI. As summarized by Dudek et al. (23), wearable sensing combined with machine learning is transforming personalized training, performance monitoring, and injury prevention, while raising challenges related to data quality, model transparency, and privacy. By incorporating attention based multimodal fusion, ADA moves toward individualized and clinically relevant assessment pipelines that extend beyond traditional kinematic only models.

### 4.2 Limitations and future directions

This pilot study involved fifteen healthy adult subjects, which limits generalizability to clinical populations or broader demographic groups. Larger, more heterogeneous cohorts are needed to validate the Ada framework’s robustness, particularly in individuals with musculoskeletal impairments. Additionally, all statistical analyses were exploratory and uncorrected for multiple comparisons; future work should incorporate confirmatory designs with appropriate error control.

Although the 17-IMU Xsens system yields detailed kinematic information, practical field applications may require fewer sensors. Prior work suggests that reduced-sensor configurations can maintain strong predictive performance (4). Future research should evaluate whether ADA generalizes effectively under minimal sensor sets or alternative wearable technologies.

The diagnostic aware component leverages sensitive subject data, highlighting the need for transparent governance, secure data handling, and adherence to emerging guidelines for ethical AI deployment in health contexts (25). Model interpretability tools such as saliency maps or feature attribution analyses should also be explored to strengthen clinical trust and explainability beyond the exploratory clustering presented here.

## 5 Conclusions

This study presented ADA, a multimodal deep learning framework that integrates wearable IMU kinematics with anthropometric and diagnostic information for subject specific assessment of squat and Romanian deadlift execution. By combining temporal feature extraction (CNN–LSTM) with attention weighted static inputs, ADA demonstrated substantially higher accuracy than kinematics only models for both movement quality classification and risk prediction.

Incorporating anthropometric measures enabled the model to account for structural variability across subjects, while diagnostic variables contributed to clinically relevant stratification. These findings indicate that subject specific information provides complementary insight beyond kinematic signals and enhances both the predictive performance and interpretability of automated biomechanical assessment.

ADA provides a scalable foundation for delivering real-time, personalized feedback in sports performance, injury prevention, and remote rehabilitation. Future work should validate the framework in larger and more heterogeneous populations, investigate reduced sensor configurations for practical field use, and develop explainable AI interfaces to support transparent clinical adoption.

## Data Availability

The raw data supporting the conclusions of this article will be made available by the authors, without undue reservation.
