# Supplementary material for: Anthropometry and diagnostic aware deep learning for exercise assessment
Source: Front Med Technol. 2026 Feb 6;7:1725661. doi: 10.3389/fmedt.2025.1725661 (PMC12920488; doi:10.3389/fmedt.2025.1725661)
Supplement: Supplementary file 1 [file Supplementaryfile1.pdf]

S1. Data Processing and Windowing

IMU-derived kinematic data were segmented using sliding temporal windows. The implemented window sizes in the analysis notebooks include 50, 100, 150, and 200 frames with a step size corresponding to approximately 50% overlap. This matches the windowing strategy described in the main manuscript.

S2. Feature Sets

The models used multi-channel IMU time-series signals combined with static anthropometric and diagnostic features. Anthropometric variables were standardized using z-score normalization prior to model input, consistent with the paper.

S3. Model Architectures

The analyses reported in the main manuscript focus on the comparison between two final deep learning models: a baseline kinematics-only model and the proposed Anthropometry and Diagnostic Aware (ADA) multimodal model. Both models were implemented using TensorFlow/Keras and shared a common temporal backbone to ensure a controlled architectural comparison.

The baseline model processed only IMU-derived kinematic sequences. Temporal feature extraction was performed using a stack of one-dimensional convolutional layers, followed by Long Short Term Memory (LSTM) layers to model sequential dependencies in the movement data. The output of the LSTM layers was passed directly to fully connected layers for binary movement quality classification (correct vs. incorrect).

The ADA multimodal model extended this baseline architecture by incorporating subject-specific anthropometric and diagnostic features through a parallel static-input branch. In this model, the kinematic branch was identical to the baseline CNN–LSTM backbone, while a separate fully connected branch processed standardized static inputs. Feature-level fusion was performed using an attention-based weighting mechanism, enabling adaptive integration of temporal kinematic representations and static subject-specific information prior to classification.

Both models produced sequence level predictions and were trained under identical optimization and validation protocols. Performance differences reported in the paper therefore reflect the contribution of anthropometric and diagnostic context rather than architectural or training discrepancies.

S4. Training and Evaluation

Training employed the Adam optimizer with learning rates on the order of 1e-4. Stratified subject wise cross validation was implemented using K-fold strategies, ensuring no subject overlap between training and testing sets. Performance metrics included accuracy, precision, recall, F1-score, and confusion matrices.

S5. Explainability and Clustering Analyses

Explainability analyses were conducted using SHAP to identify feature contributions. Latent representations were further analyzed using PCA, t-SNE, k-means, and HDBSCAN clustering. These analyses support the latent-space structure and clustering results reported in the paper.

Dataset Link:

https://github.com/drkarlareyes/Xsens-IMU-Raw-Data-Squat-RDL.git
